# The role of the cytoskeleton in biomineralisation in haptophyte algae

**DOI:** 10.1038/s41598-017-15562-8

**Published:** 2017-11-13

**Authors:** Grażyna M. Durak, Colin Brownlee, Glen L. Wheeler

**Affiliations:** 1 The Marine Biological Association of the United Kingdom, The Laboratory, Citadel Hill, Plymouth, Devon, PL1 2PB UK; 2 Department of Biology, Electron Microscopy Center, University of Konstanz, Universitätsstr. 10, D-78457 Konstanz, Germany

## Abstract

The production of calcium carbonate by coccolithophores (haptophytes) contributes significantly to global biogeochemical cycling. The recent identification of a silicifying haptophyte, *Prymnesium neolepis*, has provided new insight into the evolution of biomineralisation in this lineage. However, the cellular mechanisms of biomineralisation in both calcifying and silicifying haptophytes remain poorly understood. To look for commonalities between these two biomineralisation systems in haptophytes, we have determined the role of actin and tubulin in the formation of intracellular biomineralised scales in the coccolithophore, *Coccolithus braarudii* and in *P*. *neolepis*. We find that disruption of the actin network interferes with secretion of the biomineralised elements in both *C*. *braarudii* and *P*. *neolepis*. In contrast, disruption of the microtubule network does not prevent secretion of the silica scales in *P*. *neolepis* but results in production of abnormally small silica scales and also results in the increased formation of malformed coccoliths in *C*. *braarudii*. We conclude that the cytoskeleton plays a crucial role in biomineralisation in both silicifying and calcifying haptophytes. There are some important similarities in the contribution of the cytoskeleton to these different forms of biomineralisation, suggesting that common cellular mechanisms may have been recruited to perform similar roles in both lineages.

## Introduction

The haptophytes are a group of predominately marine algae that contribute significantly to primary productivity in the global oceans. Several lineages within the haptophytes exhibit the ability to produce biomineralised cell coverings. Most notable are the coccolithophores, which form an extracellular covering of calcified scales known as coccoliths and are major contributors to global biogenic calcium carbonate (CaCO_3_) production^1^. Whilst calcification is the primary mode of biomineralisation found in the haptophytes, a single species has been identified that is extensively silicified (*Prymnesium neolepis*, formerly *Hyalolithus neolepis*)^2^. *P*. *neolepis* produces a covering of extracellular silica scales and therefore resembles the calcifying coccolithophores to some extent, although its scales are only loosely associated with the cell and are non-interlocking^2^. There are reports indicating the presence of silicon in the cysts of other *Prymnesium* species^3,4^, although *P*. *neolepis* is the only known haptophyte capable of depositing an extensive silicified cell covering. Despite the ecological and biogeochemical importance of the haptophytes, our understanding of biomineralisation in this lineage remains limited. Comparison of the silicifying and calcifying haptophytes may provide insight into the underlying cellular mechanisms and evolutionary processes that resulted in the recruitment of these different forms of biomineralisation.

Coccolithophores produce two types of coccoliths: heterococcoliths and holococcoliths, in addition to small, non-mineralised organic scales^5^. The characteristic plate-like heterococcoliths are produced by coccolithophores in the diploid stage of their life cycle. Some species also produce the much smaller holococcoliths whilst in their haploid phase, although little is known about the mechanisms of assembly of holococcoliths. Heterococcoliths are deposited in a specialised, Golgi-derived vesicle known as the coccolith vesicles (CV)^5,6^. Coccolith production proceeds in the CV in a finely controlled process during which calcite crystals are nucleated and biomineralised on organic baseplates giving rise to complex, ornate coccoliths^7^. Previous researchers have suggested that the process of coccolith formation (coccolithogenesis) may be mechanically aided by external moulding of the CV via a fibrillar body of putatively cytoskeletal origin^8–12^. Whilst it is clear that the cytoskeleton plays a major role in biomineralisation in other algae, for example in the formation of the silica frustule of diatoms (e.g.^6,13^), little is known about the role of the cytoskeleton in biomineralisation in coccolithophores. Langer *et al*.^14^ demonstrated that treatment of the coccolithophore *Emiliania huxleyi* with colchicine, an inhibitor of microtubule polymerisation, or cytochalasin B, an inhibitor of actin polymerisation, led to a dramatic increase in the formation of malformed coccoliths. This suggests that both the actin and microtubule networks contribute to coccolith formation in *E*. *huxleyi*, although the dynamic nature of the cytoskeleton network and its interactions with the developing coccoliths remain largely uncharacterised.

The silicifying haptophyte *P*. *neolepis* has a cosmopolitan distribution and has been reported in the Atlantic, Pacific and Indian Oceans^2,15,16^. Silicification in *P*. *neolepis* shares some common elements with that of frustule formation in diatoms. In both organisms, silicification occurs in an acidic intracellular compartment, known as the silica deposition vesicle (SDV), in which silica precipitation is aided by the presence of various organic components including long chain polyamines^15,17^. In diatoms, the SDV is closely associated with the cytoskeleton. Actin plays a central role in regulating silica morphogenesis, whereas microtubules are primarily involved in regulating the size and position of the SDV^13^. Based on observations from transmission electron microscopy (TEM), Yoshida *et al*.^2^ proposed that the cytoskeleton may also play a role in regulating scale morphology in *P*. *neolepis*. However, the role of the cytoskeleton in scale formation in this silicifying haptophyte has not been characterised.

Calcification and silicification have long been thought to require highly distinct cellular mechanisms, as the precipitation of calcium carbonate and silica respectively involves very different chemistries. However, emerging evidence suggests that there may be some common mechanisms that contribute to both calcification and silicification in haptophytes and other organisms. Certain coccolithophores possess a silicon transporter resembling the SIT family of transporters found in diatoms or a novel form of SIT-like (SITL) transporter^18^. Application of the silicon analogue germanium to coccolithophores that possess SIT or SITL transporters results in severe malformations in coccolith morphology, suggesting that Si plays a direct but as yet uncharacterised role in coccolith formation^15^. The study of cellular mechanisms in the silicifying and calcifying lineages within the haptophytes may therefore contribute important information on the processes leading to the evolution of these different forms of biomineralisation both in the haptophytes, but also in eukaryotes more generally.

In order to probe the role of the cytoskeleton in these two biomineralisation systems, we used latrunculin B, which binds to actin monomers and causes F-actin depolymerisation, and nocodazole, which caps microtubules and binds to tubulin monomers, preventing their polymerisation and leading to microtubule depolymerisation at micromolar concentrations^19–22^. Biomineralisation is a complex and specialised process, but there are a number of common features between coccolithophores and *P*. *neolepis*, such as the localised precipitation of the biomineral within dedicated intracellular compartments. The role of the cytoskeleton during biomineralisation is therefore likely to involve three main elements: (1) intracellular vesicular transport of the necessary substrates, (2) active, mechanical shaping of the structures during biomineralisation, (3) exocytosis of the completed structures and their localization on the surface of the cell.

We disrupted the actin and microtubule networks in *P*. *neolepis* and in *Coccolithus braarudii*, an ecologically important coccolithophore species^15,23^. We examined the impact of cytoskeleton disruption on the arrangement of the cellular microtubule network, the morphology of the biomineralised structures, the extent of mineral deposition and the secretion of the biomineralised scales. Our results show that the actin cytoskeleton is required for correct secretion of silica scales or calcified coccoliths. In contrast the microtubule cytoskeleton plays a pivotal role in maintaining correct scale or coccolith morphology.

## Materials and Methods

### Algal cultures and growth conditions

*Coccolithus braarudii* (formerly *C*. *pelagicus* ssp. *braarudii*) (strain PLY 182 g) and *Prymnesium neolepis* (formerly *Hyalolithus neolepis*) (strain TMR5) were obtained from the Plymouth Culture Collection of Marine Microalgae and Roscoff Culture collection respectively. Cultures were maintained in aged filtered sea water with f/2 media^24^ under irradiance of 80–100 µmol s^−1^ m^−2^, with a temperature of 18 °C for *P*. *neolepis* and 15 °C for *C*. *braarudii*. *P*. *neolepis* cultures were additionally provided with 100 µM silicate. Photoperiods used were set to 18:6 h light:dark and at 12:12 light:dark for *P*. *neolepis* and *C*. *braarudii* respectively. Cell counts were carried out using a Sedgewick-Rafter cell counting chamber, with three replicate counts per sample.

### Application of cytoskeleton inhibitors

1 µM latrunculin B or 5 µg mL^−1^ nocodazole were used to disrupt either the actin or microtubule networks in all cytoskeleton inhibition experiments. The activity threshold for each cytoskeleton inhibitor was determined with a series of trial dilution experiments, using concentrations between 0.1–100 µM for latrunculin B and 0.1–10 µg mL^−1^ for nocodazole. The threshold inhibitor concentration was defined as the lowest concentration of inhibitor that resulted in the production of coccoliths or silica scales with clear morphological aberrations after 12 h, as assessed by light microscopy. Stock solutions of both inhibitors were dissolved in dimethyl sulphoxide (DMSO). We performed additional experiments to confirm that the cytoskeletal inhibitors had not caused a general disruption of cellular metabolism. The photosynthetic efficiency of photosystem II (Fv/Fm) was measured using a Z985 Cuvette AquaPen (Qubit Systems, Kingston, Canada) (Supplementary Tables S1–S2). We also assessed cell membrane integrity and cell viability of *P*. *neolepis* using 1 µM SYTOX Green (Invitrogen, Paisley, UK), which only penetrates cell membranes which are compromised (Supplementary Table S3). 25 cells from three replicates for each treatment were imaged by confocal microscopy (excitation 488 nm, emission 500–550 nm) and SYTOX Green labelling was categorised as: unlabelled, weakly labelled or strongly labelled (Supplementary Fig. S1). Chi-square tests were employed to assess if significant differences between treatments existed. The ability of *C*. *braarudii* cells to recalcify following recovery from cytoskeleton inhibitor treatment was also assessed (Supplementary Figs S2, S3).

### Immunofluorescence imaging of the microtubule network

In order to image the intracellular microtubule network in *P*. *neolepis* and *C*. *braarudii* we used a monoclonal anti-α-tubulin primary antibody from mice (M5–1–2, Sigma-Aldrich, Poole, UK) and an anti-mouse IgG secondary antibody from goat conjugated to Texas Red (#31660, Thermo Fisher Scientific, Waltham, MA). Prior to immunolabelling, *P*. *neolepis* cultures were incubated with 1 µM HCK-123 for 5 h so that the newly forming, intracellular silica scales were clearly visible. *C*. *braarudii* cells were decalcified with a Ca^2+^-free artificial sea water solution containing 25 mM EGTA (Taylor *et al*.^25^). After decalcification was complete, the ASW-EGTA solution was replaced with standard f/2 seawater media. The effect of nocodazole on the microtubule network was examined by incubating both species with 5 µg ml^−1^ nocodazole for 5 h. After treatment, cell cultures were settled onto poly-L-lysine coated glass bottom dishes, rinsed three times with ASW and fixed for 10 min in an ASW solution containing 2% glutaraldehyde and 1.7% BSA (bovine serum albumin). Samples were subsequently washed three times with a solution of ASW/1.7% BSA with 0.5% glutaraldehyde and incubated for 10 min in 0.05% Triton X-100 in ASW. The samples were then washed three times with ASW/1.7% BSA and incubated for a further 20 min. After the fixing procedure was complete, samples were incubated overnight in a 1/50 dilution of the primary anti-α-tubulin antibody, washed 3x with ASW/1% BSA and then incubated in a 1/150 dilution of the secondary Texas Red-conjugated antibody for 2.5 h. Cells were then washed a final three times with ASW/1.7% BSA prior to confocal microscopy. Three independent experiments were carried out, with each treatment performed in triplicate to confirm the consistency of the microtubule labelling patterns observed.

Immunofluorescence in fixed cells of *P*. *neolepis* and *C*. *braarudii* was viewed using a LSM 510 confocal laser scanning microscope (Zeiss, Cambridge, UK). Texas Red was excited at 543 nm, with emission at 575–625 nm. HCK-123 was viewed with excitation at 488 nm and emission 500–550 nm. Calcite was imaged using reflectance, with excitation at 633 nm and a short pass emission filter at 685 nm. Incubation with secondary antibodies alone was used to provide images of non-specific staining (Supplementary Figs S4–S5).

### Confocal microscopy of live cells

The parameters used for live cell imaging were identical to those used for immunofluorescence imaging, with chlorophyll autofluorescence additionally collected between 650–710 nm after excitation at 488 nm. 1 µM Lysotracker Yellow HCK 123 was added to *P*. *neolepis* cultures 2 h after the treatment with cytoskeleton inhibitors for the duration of the treatment. To determine the dimensions of newly produced silica scales, the maximal diameter of individual scales labelled with HCK-123 was measured for each treatment. The variability and difference in scale length were assessed statistically with a two sample Kolmogorov-Smirnov test and a *t-*test (Statsoft: Statistica) respectively. To determine the number of intracellular scales in *P*. *neolepis*, cells were lysed using a Mai Tai pulsed multiphoton infra-red laser at 740 nm (Spectraphysics, Santa Clara, CA).

### Flow cytometry analysis of silica scale production in *P*. *neolepis*

To assess the effects of cytoskeleton inhibition on silica scale production, *P*. *neolepis* cells treated with cytoskeleton inhibitors and labelled with HCK-123 were harvested by centrifugation and washed three times with 0.1 M Tris buffer at pH 8.00 containing 0.1 M EDTA and 2% SDS to remove cellular debris. The scales were then washed three times with deionised water and analysed with an Accuri C6 flow cytometer recording side scatter and green fluorescence (SSC vs. FL1A). This procedure was replicated three times in five independent experiments. Differences between treatments were determined with a Student’s *t*-test for independent samples (Statsoft: Statistica).

### Differential Interference Contrast (DIC) microscopy of *C*. *braarudii*

In order to assess the effects of cytoskeleton inhibitors on coccolith production in *C*. *braarudii*, we first decalcified cultures by adding HCl to decrease the pH to pH 4.00 for 3 min. The pH was then returned to 8.20 with NaOH. Low pH was used for de-calcification in these experiments as the proportion of cells recalcifying was much higher and more consistent than with the EGTA method. The cells were examined by light microscopy to ensure that they were fully decalcified and then settled onto poly-L-lysine coated glass bottom dishes in 1 ml aliquots. After the cells had settled, an additional 2 ml of f/2 media in seawater was added. Samples were imaged by DIC microscopy after a 24 h incubation period with the cytoskeleton inhibitors. Images were acquired using a Nikon Eclipse Ti microscope equipped with a Photometrics Evolve EM-CCD camera. To ensure that cells were still viable after cytoskeleton disruption, the inhibitors were removed after 24 h incubation and cells were supplied with fresh f/2 media in seawater. Cell recovery was monitored at 24 h intervals for a further 48 h (Supplementary Figs S2, S3).

### Scanning electron microscopy (SEM) of coccoliths produced by *C*. *braarudi*

*C*. *braarudii* cells were treated with cytoskeleton inhibitors as described above for DIC imaging. After the 24 h incubation period, the cells were harvested by centrifugation and cellular pellets were mixed with 1 mL of 10–15% NaOCl (Sigma-Aldrich) for 1 h to remove cellular debris. Samples were then washed three times with deionised water adjusted with NH_4_OH to pH 10.00. The cleaned coccoliths were then filtered onto a 0.22 µm polycarbonate filter and sputter coated with gold-palladium for electron microscopy examination. Samples were imaged with a JEOL JSM-7001F Field Emission Microscope and JEOL JSM-6610LV Scanning Electron Microscope at 15 kV accelerating voltage. We analysed 100 coccoliths per replicate with three replicates per treatment (n_total_ = 300) for the control and nocodazole treatments. To assess the effects of microtubule inhibition we assigned the coccoliths into three categories: complete, partially formed/slightly malformed and malformed coccoliths.

## Results

### Immunofluorescence microscopy of microtubule network in *P*. *neolepis* and *C*. *braarudii*

**Figure 1:**
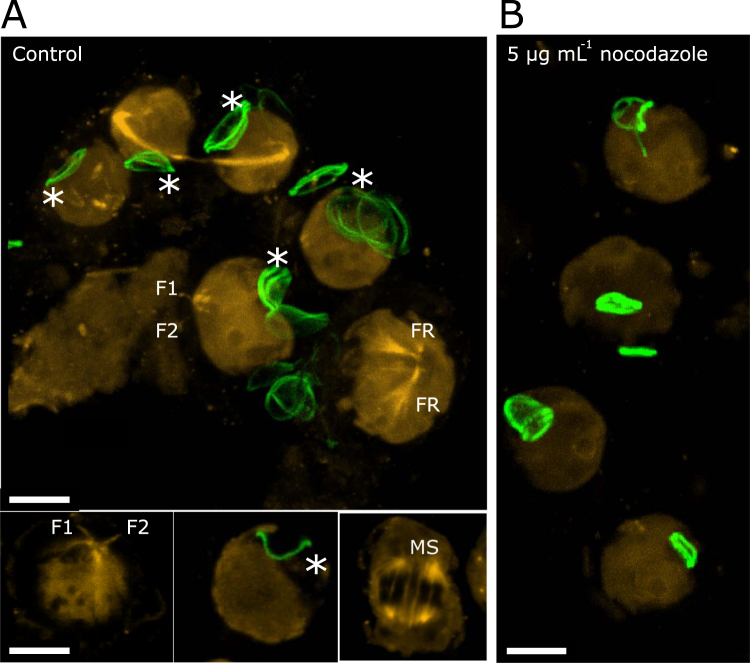
Immunofluorescence imaging of microtubule networks in *P*. *neolepis*. (**A**) *P*. *neolepis* cells labelled with an anti-tubulin antibody indicating the extent of the microtubule networks (yellow). The diffuse non-specific background labelling of the cell body is also present. Specific microtubule structures can be observed such as the two short flagella (F1 and F2), flagellar roots (FR), mitotic spindles (MS) and a large microtubule cable between the dividing cells. Newly formed silica scales were labelled with HCK-123 (green), intracellular scales with the concave side facing externally are marked with an asterisk. A 3D projection from a Z-stack is shown (large image, upper panel), alongside individual confocal slices (lower). (**B**) *P*. *neolepis* cells after treatment with 5 µg mL^−1^ nocodazole for 5 h. The tubulin networks have been extensively disrupted and the silica scales are smaller with an altered morphology. A 3D projection of a confocal Z-stack is shown, scale bars correspond to 5 µm.

In order to investigate the role of the cytoskeleton in haptophyte biomineralisation, we first examined the extent and localisation of the microtubule network in *P*. *neolepis* and *C*. *braarudii* using immunofluorescence microscopy. Although there was a degree of background autofluorescence (caused by fixation with glutaraldehyde), we were able to observe several larger microtubule structures in both organisms. The most prominent features of the microtubule network in *P*. *neolepis* were associated with the flagella and the haptonema. *P*. *neolepis* has two short flagella (<1 µm) and a longer non–coiling haptonema (12–18 µm)^2^. These structures were clearly visible in many cells, along with the basal bodies and the flagellar roots (Fig. 1A). In cells undergoing cell division, we were able to observe mitotic spindles and a large microtubule cable between the cells present during cytokinesis. The silica scales of *P*. *neolepis* are formed in an acidic silica deposition vesicle, enabling selective labelling of newly formed scales with fluorescent dyes that partition into acidic compartments^15,26^. We were therefore able to simultaneously observe the position of intracellular silica scales with the microtubule network. We did not observe any intimate associations between the microtubules and the silica scales in *P*. *neolepis*, although we cannot rule out fine scale associations that are beyond the resolution of our approach. Treatment of *P*. *neolepis* with 5 µg mL^−1^ nocodazole for 5 h resulted in significant disruption of the microtubule network with only some remnants of the flagellar roots, the haptonema and the mitotic spindles discernible (Fig. 1B).

In agreement with previous observations^2^, we found that all intracellular scales are positioned with the concave side facing the plasma membrane. All extracellular scales exhibit the reverse orientation, with the concave side facing the cell, indicating that the scales must be reversed at some point during the secretion process. As these processes are likely to be coordinated by the cytoskeleton, we used time-lapse video microscopy to further examine scale secretion and orientation. We found that the intracellular scales are secreted with the concave side facing initially outwards. The scale is then inverted whilst outside the cell, indicating that reorientation of the scale occurs after secretion (Supplementary Fig. S6).

**Figure 2:**
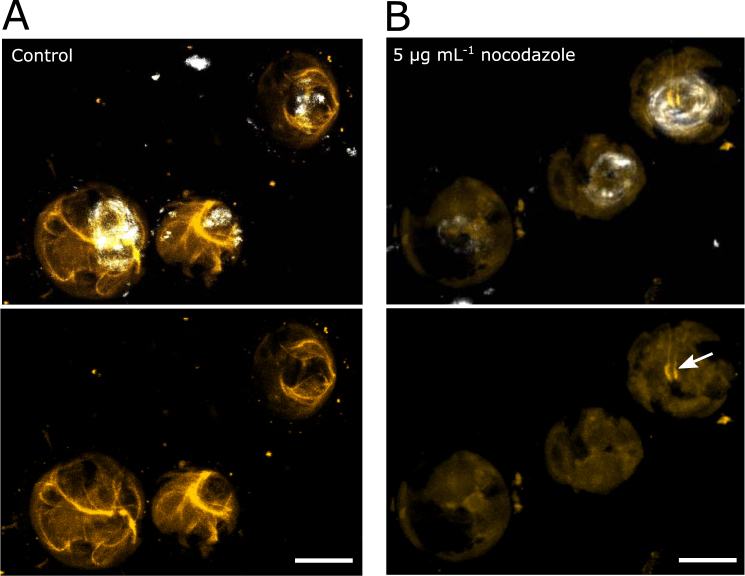
Immunofluorescence imaging of microtubule networks in *C*. *braarudii*. (**A**) *C*. *braarudii* cells labelled with an anti-tubulin antibody indicating the extent of the microtubule networks (yellow). There is an extensive network of microtubules throughout the cell, with an association of microtubules around the intracellular coccolith. Cells were decalcified to remove extracellular coccoliths prior to imaging. Intracellular coccoliths were viewed by reflectance microscopy (white). The lower image shows the microtubule networks without the coccolith overlay for clarity. (**B**) *C*. *braarudii* cells after treatment with 5 µg ml^−1^ nocodazole for 5 h. Remnants of tubulin aggregations can be observed close to the intracellular coccoliths (arrows). Scale bars correspond to 5 µm. All images represent 3D projections of Z-stacks obtained by confocal microscopy.

Immunofluorescence microscopy of the coccolithophore *C*. *braarudii* revealed an extensive microtubule network, in contrast with the limited structures observed in *P*. *neolepis*. TEM studies of *C*. *braarudii* demonstrated the presence of bundles of microtubules that extend from the plasma membrane into the centre of the cell and can be found in close proximity with the coccolith vesicle^25^. These microtubule bundles were clearly observed in our immunofluorescence images (Fig. 2A). In many cells, the bundles of microtubules form an ovoid structure surrounding the maturing coccolith. This close association suggests that the microtubule network may play a direct role in coccolith formation and/or secretion. After treatment with nocodazole, virtually no microtubule structures could be observed in *C*. *braarudii*, indicating that the microtubule network had been extensively disrupted (Fig. 2B).

Actin is also very likely to be associated with the organisation and secretion of the biomineralised scales. We made repeated attempts to examine the actin network in both *P*. *neolepis* and *C*. *braarudii* using established protocols employed in other algae (e.g. FITC-phalloidin^27^), however, we were unable to successfully visualise actin structures within these species. Our direct observations of the cytoskeleton are therefore restricted to the microtubule network.

### Effects of cytoskeleton disruptors on silica scale morphogenesis in *P*. *neolepis*

**Figure 3:**
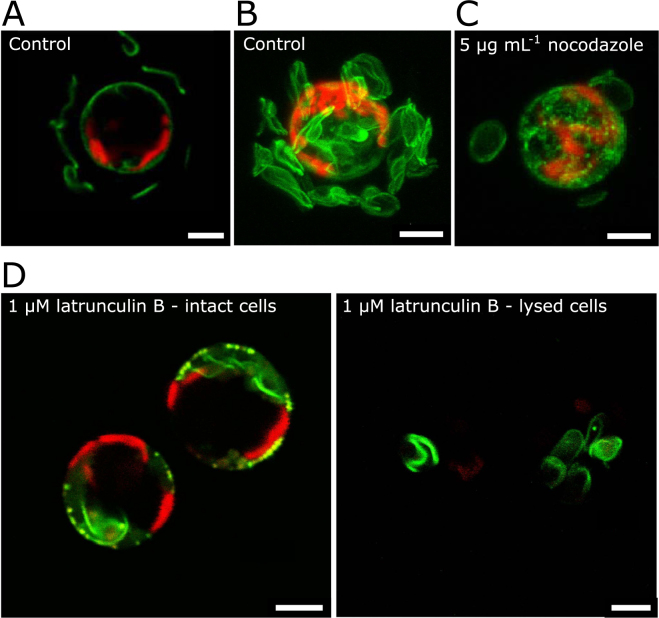
The impact of cytoskeleton disruption on scale formation in *P*. *neolepis*. (**A**) Live cell imaging of a control *P*. *neolepis* cell loaded with HCK-123 for 24 h, viewed by confocal microscopy. HCK-123 was incorporated into newly formed silica scales and abundant extracellular scales can be observed. Chlorophyll autofluorescence is shown in red. (**B**) A 3D projection of a control cell demonstrating the extensive coverage of silica scales. (**C**) A 3D projection of a *P*. *neolepis* cell treated with nocodazole (5 µg mL^−1^, 24 h) exhibiting reduced coverage of silica scales. Individual scales are noticeably rounder and smaller. (**D**) A representative *P*. *neolepis* cell following treatment with 1 µM latrunculin B for 24 h indicates the absence of extracellular scales (left). Scales accumulate intracellularly and can be clearly observed following cell lysis (right). Scales produced during the treatment were labelled with HCK-123 (green), chlorophyll autofluorescence is shown in red. A total of 87 individual cells were examined (16–27 cells examined in four replicate treatments). Scale bars correspond to 5 µm.

As the fluorescent dye HCK-123 is only incorporated into newly produced silica scales in *P*. *neolepis*, we were able to assess the effect of cytoskeleton disruption on the production and secretion of the silica scales. Control cells treated with HCK-123 for 24 h accumulate multiple fluorescently-labelled extracellular scales (Fig. 3A,B). In contrast, cells treated with 1 µM latrunculin B exhibit no extracellular fluorescent scales after 24 h, but do accumulate fluorescent scales inside the cell. (Fig. 3D). These intracellular scales could be clearly observed after cell lysis, with up to 8 scales observed per latrunculin-treated cell compared to the 2–3 intracellular scales found in control cells. The intracellular silica scales produced during the latrunculin B treatment did not show any obvious malformations. This suggests that the principal defect caused by actin disruption relates to the secretion of the scale rather than its formation.

**Figure 4:**
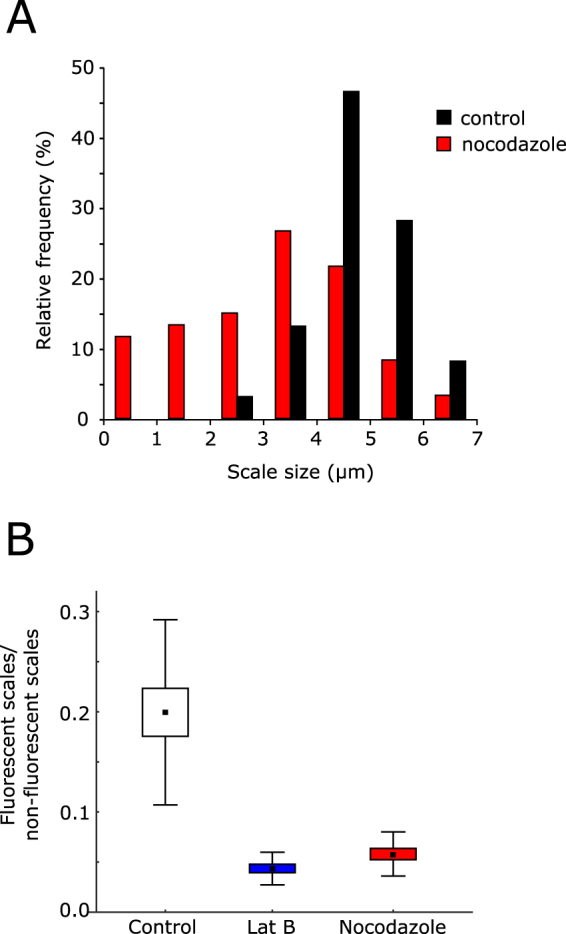
Cytoskeleton disruption results in reduced production of silica scales. (**A**) Frequency histogram demonstrating the effect of nocodazole (5 µg mL^−1^, 24 h) on the size of silica scales produced by *P*. *neolepis*. The maximal diameter of HCK-123 labelled scales viewed by confocal microscopy was measured for nocodazole-treated and control cells (n = 60 scales). (**B**) Box-whisker plots demonstrating production of HCK-123 labelled scales in cells treated cytoskeleton inhibitors. The ratio of fluorescent (newly-produced scales) to non-fluorescent (existing scales) scales was measured by flow cytometry. Cells were treated with 1 µM latrunculin B or 5 µg mL^−1^ nocodazole for 24 h (n = 5).

In contrast to latrunculin treatment, cells treated with 5 µg mL^−1^ nocodazole to disrupt the microtubule network exhibited multiple labelled extracellular scales after 24 h (Fig. 3C). However, the morphology of the scales following nocodazole treatment was very different to control cells, with the scales appearing to be rounder and smaller. We performed quantitative analysis of scale morphology using confocal microscopy to measure individual fluorescently-labelled scales. This approach ensured that only newly produced scales were analysed, as existing scales were not labelled. Although other imaging techniques, such as scanning electron microscopy, would allow greater resolution of scale morphology, differentiation of the newly-produced scales from the large background of scales produced prior to treatment would not be possible. Our analyses indicated that the mean maximal diameter of control scales was 4.69 ± 0.89 µm, whereas scales produced by nocodazole-treated cells were significantly smaller at 3.21 ± 1.52 µm (*t* = −5.83, p < 0.05, n = 60). The difference in the mean scale diameter in samples treated with nocodazole was the result of the production of abnormally small scales (0.5–2 µm) and a decrease in the production of scales above 4 µm length (Fig. 4A). To assess whether nocodazole treatment also inhibited the number of scales produced, we developed a flow cytometric assay to quantify scale production. Scales were extracted from cells and cleaned for flow cytometry using a 2% SDS buffer. It is important to note that this method lysed the cells and therefore included both intracellular and extracellular scales. To measure new scale production, we determined the proportion of fluorescent scales (i.e. scales produced during treatment) relative to non-fluorescent scales (scales produced in culture before fluorescent labelling). Changes in this ratio will therefore reflect the number of scales produced. As expected, cells treated with latrunculin B exhibited a dramatic decrease in the production of new scales (Fig. 4B) (t = 12.387, p < 0.05, difference between means = 0.157). Nocodazole treatment also resulted in a significant decrease in the number of scales produced (*t* = 0.142, p = <0.05, difference between means = 10.754). The rate of scale production also differed significantly between latrunculin B and nocodazole treated samples (*t* = 2.097, p < 0.05, difference between means = 0.015) corroborating the direct observations by microscopy.

### Effects of cytoskeleton disruptors on biomineralisation in *C*. *braarudii*

**Figure 5:**
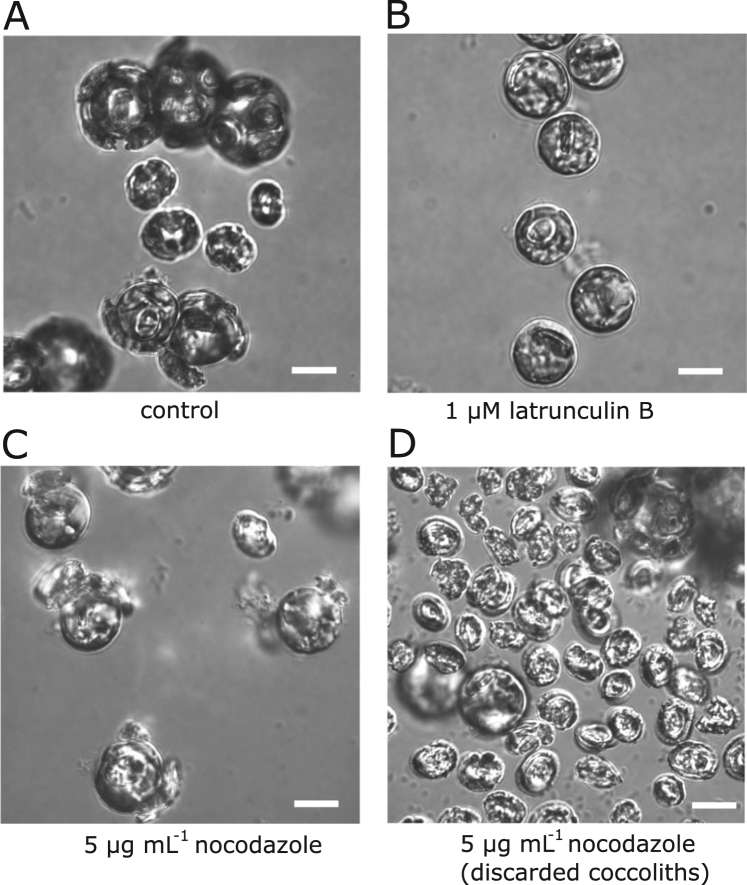
The impact of cytoskeleton disruption on coccolith production in *C*. *braarudii*. *C*. *braarudii* cells viewed by differential interference contrast (DIC) microscopy. Cells were decalcified and allowed to recalcify for 24 h in the presence of the cytoskeleton inhibitors latrunculin B (1 µM) or nocodazole (5 µg mL^−1^). (**A**) Untreated cells exhibit extensive recalcification within this period (control). (**B**) No coccolith formation was observed following treatment with latrunculin B. (**C**) Extensive coccolith production was observed in nocodazole-treated cells, although few cells exhibited intact coccospheres and many discarded coccoliths were observed. (**D**) Close examination of discarded coccoliths from the nocodazole-treated sample indicated that many were significantly malformed. The images are representative of three experimental replicates for each treatment. Scale bars correspond to 10 µm.

We next examined whether disruption of the cytoskeleton had similar impacts on the production of coccoliths in *C*. *braarudii*. To specifically assess the impact of the treatments on newly formed coccoliths, we decalcified *C*. *braarudii* cells prior to treatment and then allowed the cells to recalcify for 24 h in the presence of the inhibitors before viewing them by differential interference contrast microscopy (DIC) (Fig. 5A). Disruption of the actin cytoskeleton in *C*. *braarudii* by treatment with 1 µM latrunculin B for 24 h had a major impact on calcification. We observed a complete inhibition of coccolith exocytosis, with no cells exhibiting external coccoliths (Fig. 5B). A single partially-formed intracellular coccolith was visible in many cells, although these were likely present prior to the treatment and remained in the cell in the absence of coccolith exocytosis. We also observed a number of cells which did not have any intracellular coccoliths, suggesting that latrunculin B treatment may also have inhibited coccolith formation in addition to secretion.

Disruption of the microtubule network by 5 µg mL^−1^ nocodazole did not inhibit coccolith secretion, as many cells exhibited external coccoliths (Fig. 5C). However, the coccosphere of nocodazole-treated cells after 24 h was less complete than in control cells and there were many discarded coccoliths that had not integrated into the coccosphere (Fig. 5D). Closer examination of the coccoliths secreted by nocodazole-treated cells indicated that many were malformed, which may have affected their ability to integrate properly into the coccosphere. These malformations were examined in greater detail using scanning electron microscopy (SEM).

### SEM analysis of coccolith malformations in *C*. *braarudii* induced by cytoskeleton disruption

**Figure 6:**
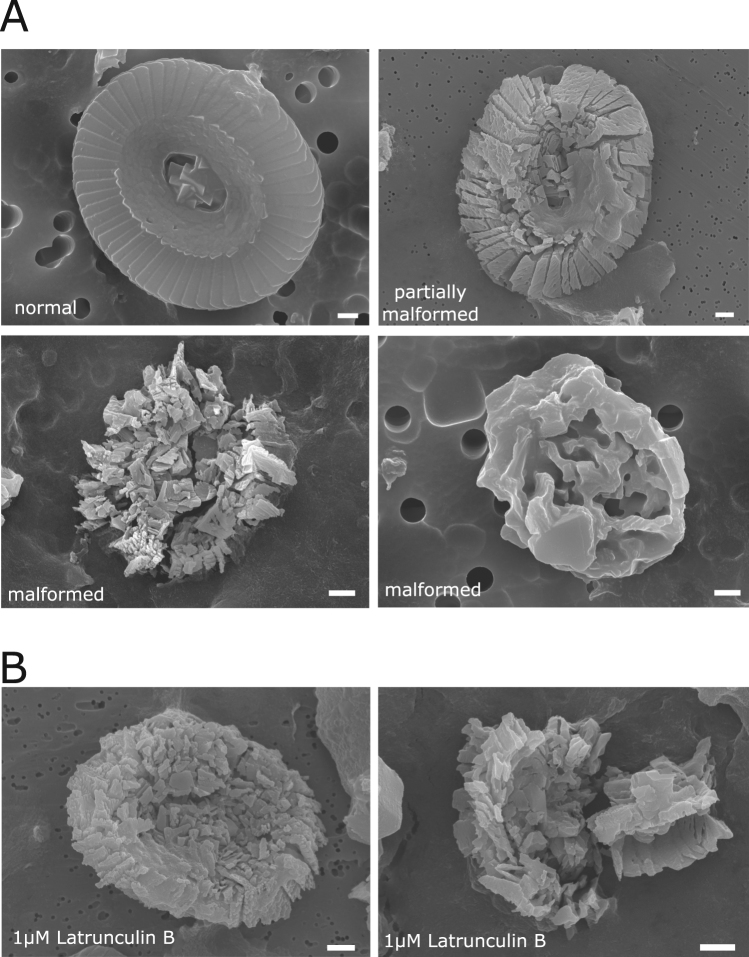
Coccolith malformations induced by cytoskeleton disruption in *C*. *braarudii*. (**A**) Examples of *C*. *braarudii* coccoliths used for ranking morphological aberrations. Coccoliths from control or nocodazole treated cells (described in Fig. 5) were viewed by SEM. Coccoliths were ranked as normal, partially malformed/incomplete or malformed. (**B**) Examples of coccoliths observed after the 24 h treatment with 1 µM latrunculin B. As extracellular liths were not observed in latrunculin B treated cells, the coccoliths observed by SEM were presumed to derive from intracellular coccoliths. Scale bars correspond to 1 µm.

Coccoliths from cells following the 24 h treatments detailed above were collected and prepared for the SEM analysis. The pelleted cultures were treated with sodium hypochlorite, which removes organic material from the coccoliths and so the samples included both extracellular and intracellular coccoliths. Coccoliths imaged by SEM were categorised into three groups based on their morphology: (1) normal, representing fully formed coccoliths with the correct morphology, (2) partially malformed/incomplete coccoliths with slight structure deformation and (3) malformed, heavily distorted coccoliths with little recognisable structure of a mature coccolith (Fig. 6). Coccoliths assigned to each of these three morphological classes were enumerated for control and nocodazole treatments. After undergoing decalcification, *C*. *braarudii* cells will often initially produce 1–2 malformed coccoliths^25^ and so the proportion of malformed coccoliths in recalcifying cells was higher than usually observed for healthy cells with intact coccospheres. However, there was a very clear and significant increase in the malformations caused by nocodazole treatment, with only 27.7% of coccoliths examined appearing to have normal morphology compared to 65.7% in control cells (Table 1).

**Table 1:** Coccolith malformations in *C*. *braarudii* cells after disruption of microtubule network.

Coccolith category	control (%)	nocodazole 5 µg mL^−1^ (%)	p
Normal	65.67	27.70	<0.001
Partially malformed	18.67	28.00	<0.001
Malformed	15.67	44.30	<0.001

Coccoliths were viewed by SEM and categorised according to their morphology. Statistically significant differences were determined by a Chi-squared analysis. n = 300 coccoliths for each treatment.

We did observe some coccoliths in sample preparations from latrunculin B treated cells, even though this treatment completely blocked coccolith exocytosis. We therefore presume that these coccoliths represent intracellular coccoliths that were most likely partially formed prior to the treatment. Although it was not possible to determine if the coccoliths continued to form during the treatment with latrunculin B, their appearance is severely malformed (Fig. 6B), suggesting that there may have been some limited calcification after disruption of the actin network.

## Discussion

The most important functions of the cytoskeleton in eukaryotes include the maintenance of cell shape, transport of vesicles and molecules, cell movement, cell division, orientation of the organelles and cytoplasmic streaming^28–30^. Both actin filaments (F-actin) and microtubules are involved in these processes and frequently interact, creating intracellular tracks used for molecular transport, driven by motor proteins such as the microtubule-associated kinesins and dyneins or the F-actin linked myosins^31–33^. Therefore, cytoskeleton disruption is likely to affect biomineralisation via three major cytoskeleton-dependent processes: (1) intracellular transportation, (2) direct shaping of the biomineral structure, (3) exocytosis. Disruption of the former two roles may result in defects in morphology, whereas the latter could lead to the absence of extracellular scales and/or the accumulation of intracellular scales.

### Effects of microtubule inhibition on biomineralisation

The microtubule network was extensively disrupted by the addition of nocodazole in both *P*. *neolepis* and *C*. *braarudii*, indicating that this treatment was likely to have a major impact on microtubule-dependent processes. Both cell types were able to produce extracellular scales after nocodazole treatment for 24 h, although the newly formed scales exhibited extensive malformations in both species. Intracellular scales exhibiting extensive malformations can be observed after 5 h in *P*. *neolepis*, indicating that microtubule disruption has a rapid effect on scale production. Malformations were not apparent in the intracellular coccoliths of *C*. *braarudii* after 5 h, although the extracellular coccoliths were not observed. The differences between species may also be due to the lower rate of coccolith formation, as only 1–2 coccoliths are usually produced within 5 h (Taylor *et al*.^25^; Durak *et al*.^15^). Our results suggest that the microtubule network is important for scale formation in both species, but there is no clear evidence for a role in scale secretion.

Defects in scale morphology are likely to arise from either disruption of the supply of precursors or the direct regulation of morphology. As microtubule-associated kinesins and dyneins are involved in transportation of membranous and non-membranous molecules, vesicles, vacuoles and endoplasmic reticulum (ER)^34^, damage to the microtubule network could result in an interruption in the supply of substrates to the SDV or the coccolith vesicle. Microtubule disruption could also hinder ER distribution as well as Golgi-derived secretion which are both heavily involved in biomineralisation in coccolithophores^1,35,36^.

Immunofluorescence imaging of the microtubule network provided some evidence for an association between the microtubules and the coccolith vesicle in *C*. *braarudii*. The arrangement of the microtubule bundles, resembling a contractile flagellar root, could contribute to secretion of the mature coccolith^25^, although we did not directly observe any defects on secretion. The altered morphology of the coccoliths following nocodazole treatment suggests that these microtubule bundles may contribute to maintaining the size and shape of the developing coccolith. This is consistent with a previous study in the coccolithophore *E*. *huxleyi*, which demonstrated that treatment with colchicine lead to extensive coccolith malformations^14^. The severe distortion of the coccolith structure in *C*. *braarudii* indicates that microtubules may be involved in guiding the growth of the calcite crystals by providing mechanical control and delimiting the structure as it grows in the CV. Whilst we did not observe direct interactions between the microtubule bundles and the CV, it is possible that they play a dual role in coccolith formation, both in shaping the developing coccolith as well as acting to supply the necessary components to the growing structure.

### Effects of actin inhibition on biomineralisation

Our findings indicate that actin plays an important role in biomineralisation in *P*. *neolepis* and *C*. *braarudii*, although it was not possible to visualise the actin network in either species. Treatment with latrunculin B resulted in a complete absence of extracellular scales in both species, suggesting that exocytosis of the biomineralised structures in *P*. *neolepis* and *C*. *braarudii* is an actin-dependent process. This is consistent with the well-characterised role for myosin-driven transport of secretory vesicles along actin cables in plant, animal and fungal cells^31,37–39^. The accumulation of intracellular scales in *P*. *neolepis* indicates that silicification continued to occur after disruption of the actin network. Therefore, formation of the silica scale does not appear to be actin dependent. Intracellular coccoliths did not accumulate in *C*. *braarudii*, following latrunculin B treatment but as this species usually produces a single intracellular coccolith at any one time, there are likely to be feedback processes that prevent the accumulation of further coccoliths prior to secretion of the mature coccolith. As the intracellular coccoliths from latrunculin-treated cells were severely malformed, it is likely that an intact actin network is required for proper coccolith morphology.

The effects of the disruption of F-actin in *C*. *braarudii* with latrunculin differed from those observed in another coccolithophore, *E*. *huxleyi* after treatment with cytochalasin B^14^. In that study, cytochalasin B did not stop coccolithogenesis but resulted in the production malformed and incomplete coccoliths, with only 15% of coccoliths having normal morphology compared to 87% in the control^14^. The reasons for these differing responses could stem from the mode of action of the inhibitors used. Cytochalasin B does not inhibit monomer addition or filament annealing at the pointed end of actin filaments and promotes focal accumulations of F-actin rather than completely depolymerising F-actin as is the case with latrunculin^20,40–42^. The nature of the two treatments also differed, as cytochalasin B was applied to calcified *E*. *huxleyi* cells for several days at a concentration that enabled the cells to continue to divide and grow. Therefore, it is likely that some residual F-actin was present in the cytochalasin B-treated *E*. *huxleyi* cells. Additionally, these two species exhibit some mechanistic differences in coccolith formation^15,43^, which may influence the requirement for cytoskeletal components during biomineralisation.

### Considerations of the broader effects of cytoskeleton disruptors

Both nocodazole and latrunculin B have well-characterised specific modes of action in disrupting the cytoskeleton and have been routinely used at similar concentrations in many different organisms^44–47^. Although these inhibitors cause extensive disruption to the cytoskeleton, their effects are readily reversible and the inhibitors are not generally toxic to the cell when used at appropriate concentrations for short incubation periods. We have used immunofluorescent microscopy to demonstrate that nocodazole is an effective disruptor of the microtubule network in haptophytes. However, disruption of the cytoskeleton may have many impacts on cell physiology and it is important to consider this when evaluating the effect of these inhibitors on the biomineralisation process. In particular, significant disruption of actin and microtubule networks will exert a strong influence on cell division, slowing or stopping the growth of unicellular organisms which could lead to indirect impacts on the biomineralisation process. We did not directly monitor growth during our treatments, although our measures of culture health (e.g. photophysiology, cell viability and ability to re-calcify) suggested that the physiology of either species was not largely disrupted. As nocodazole and latrunculin B had very distinct effects on biomineralisation in both species, we suggest that these effects are most likely due to specific disruption of the cytoskeleton and are not generic effects on biomineralisation due to slow growth or poor culture health. Moreover, the phenotypes of nocodazole- or latrunculin B-treated *C*. *braarudii* cells are distinct from coccolith malformations induced by other stressors that may affect growth, such as phosphate limitation or elevated temperatures^48,49^.

### Differential role of cytoskeleton in silicification in haptophytes and diatoms

Both actin and tubulin cytoskeleton components are heavily involved in diatom frustule morphogenesis since they participate in mechanical moulding of the SDV^13,50^. Actin has been identified as the major component involved in control over silica deposition during diatom valve morphogenesis at the micro- and meso-scale as well as the overall valve shape, and positioning of structures such as the raphe^13,50,51^. In contrast, in the haptophyte, *P*. *neolepis*, actin appears to be primarily involved in exocytosis rather than formation of the silica scales, as we observed an accumulation of intracellular scales without defects in morphology upon treatment with latrunculin B. The microtubule network in diatoms is used for localisation of the SDV formation site and so frustule formation cannot be initiated if the microtubules are disrupted^50^. As the microtubules are also responsible for strengthening and positioning of specific parts of the frustule, disruption of the microtubules during valve morphogenesis causes severe malformations^50^. Although no intimate SDV-microtubule associations were observed with immunofluorescence staining in *P*. *neolepis*, microtubules were shown to play a role in controlling the overall shape of silica scales, possibly by delimiting the shape of the SDV. Disruption of microtubules in *P*. *neolepis* caused morphological alteration of silica scale formation which produced smaller rounded scales. Silica scale production was decreased significantly by disruption of the microtubules but not completely inhibited, suggesting that formation of the SDV and the initiation of scale production can continue to a limited degree.

In summary, our results show that the cytoskeleton plays a central role in both silicification and calcification in haptophytes. Interestingly, there were some striking similarities in the effects of latrunculin and nocodazole on the formation of both types of biomineralised scales. Our results suggest a conserved role for actin in scale secretion and a role for the microtubule network in scale formation and morphology. Biomineralisation appears to have evolved in these lineages independently, significantly after the divergence of the calcihaptophycidae and the prymnesiales (c. 300 MYA)^52^. *C*. *braarudii* and *P*. *neolepis*, show important mechanistic differences in biomineralisation, such as different origin of the SDV vs. Golgi-derived CV, different formation and exocytosis site and the absence of an organic baseplate in *P*. *neolepis* ^15^, indicating that many of the underlying cellular mechanisms are not homologous. Nevertheless, whilst the underlying chemistries of silicification and calcification are fundamentally different and the biomineralisation processes have therefore evolved independently, it seems likely that some common cellular mechanisms have been recruited to perform similar roles in both forms of biomineralised haptophytes.

## Electronic supplementary material

Supplementary information
