# Supplementary material for: The role of the cytoskeleton in biomineralisation in haptophyte algae
Source: Sci Rep. 2017 Nov 13;7:15409. doi: 10.1038/s41598-017-15562-8 (PMC5684398; doi:10.1038/s41598-017-15562-8)
Supplement: Supplementary file 1 — Supplementary information [file 41598_2017_15562_MOESM1_ESM.doc]

**The role of the cytoskeleton in biomineralisation in haptophyte algae**

Grażyna M. Durak, Colin Brownlee, Glen L. Wheeler

**Supplementary Information**

Supplementary Figures S1-S6

Supplementary Tables S1-S3

**Supplementary Figures**


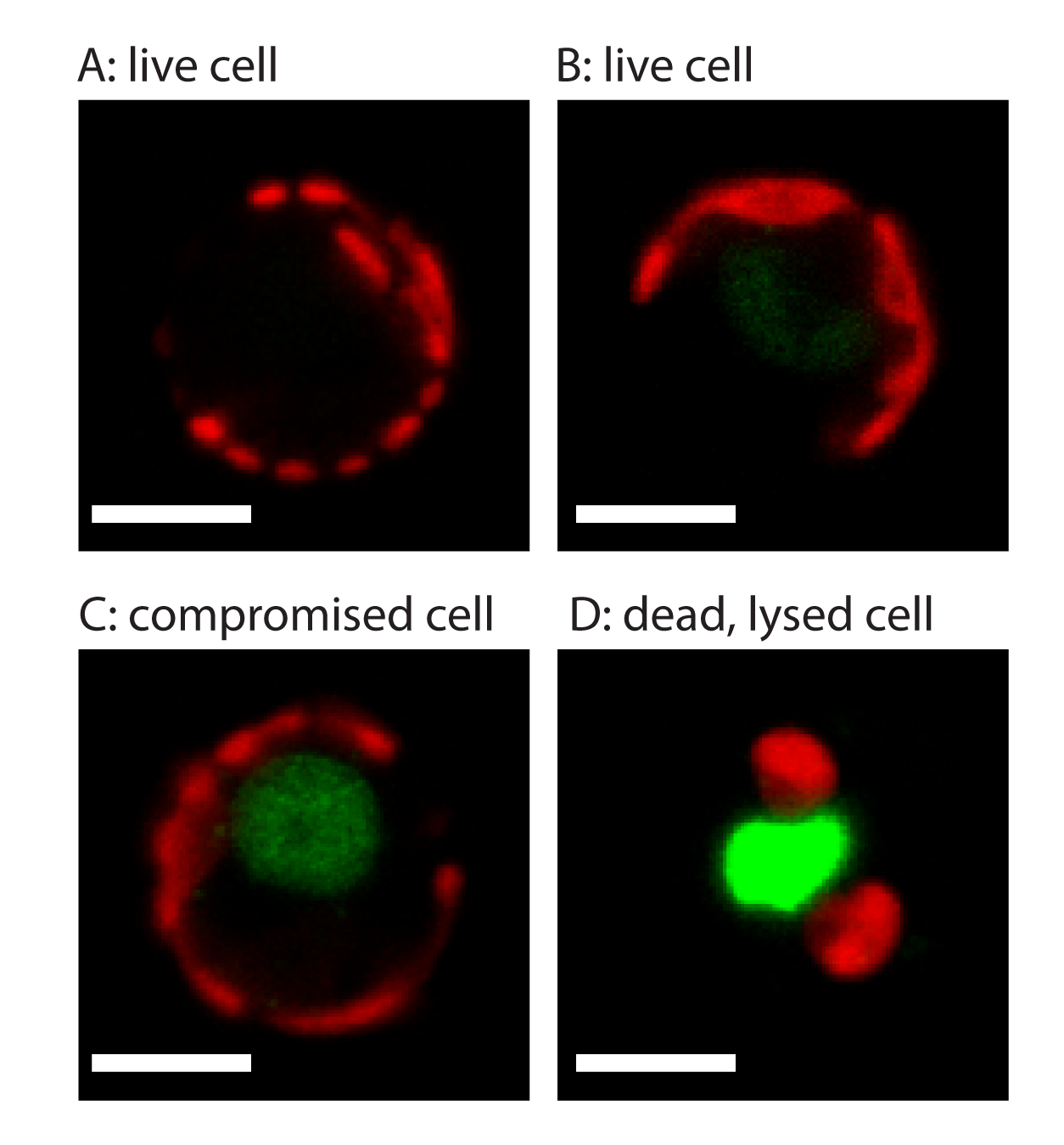


**Supplementary Figure S1: SYTOX Green labelling of *P. neolepis* cells.**

Representative confocal microscopy images of SYTOX Green labelled *P. neolepis* cells demonstrating three categories of cell labelling. A) unlabelled live cell, B) weakly labelled live cell, C) strongly labelled cell, suggesting that membrane integrity may be compromised. D) strongly labelled, partially lysed cell. Scale bar = 5 µm. Chlorophyll autofluorescence is shown in red.


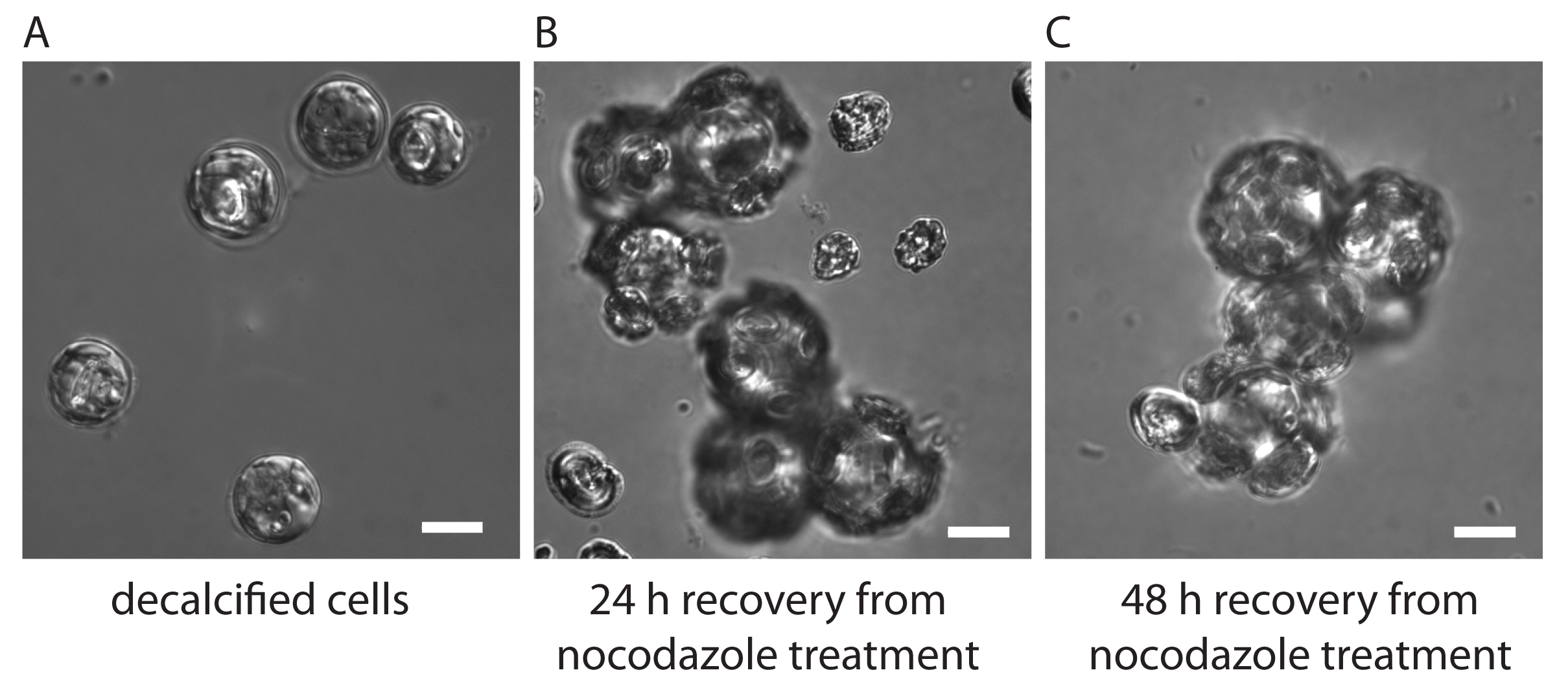


**Supplementary Figure S2: *C. braarudii* recovery from nocodazole treatment**

DIC microscopy of *C. braarudii* cells demonstrating recovery from nocodazole treatment. Cells were initially decalcified and then treated with 5 µg mL^-1^ nocodazole for 24 h. A). Cells shown directly after decalcification. B) Cells 24 h after removal of nocodazole. C) 48 h after removal of nocodazole. The cells become fully calcified after the removal of nocodazole, indicating that the disruption of the microtubule network does not lead to a general disruption of cell physiology or an irreversible defect in coccolith formation. Scale bar = 5 µm.


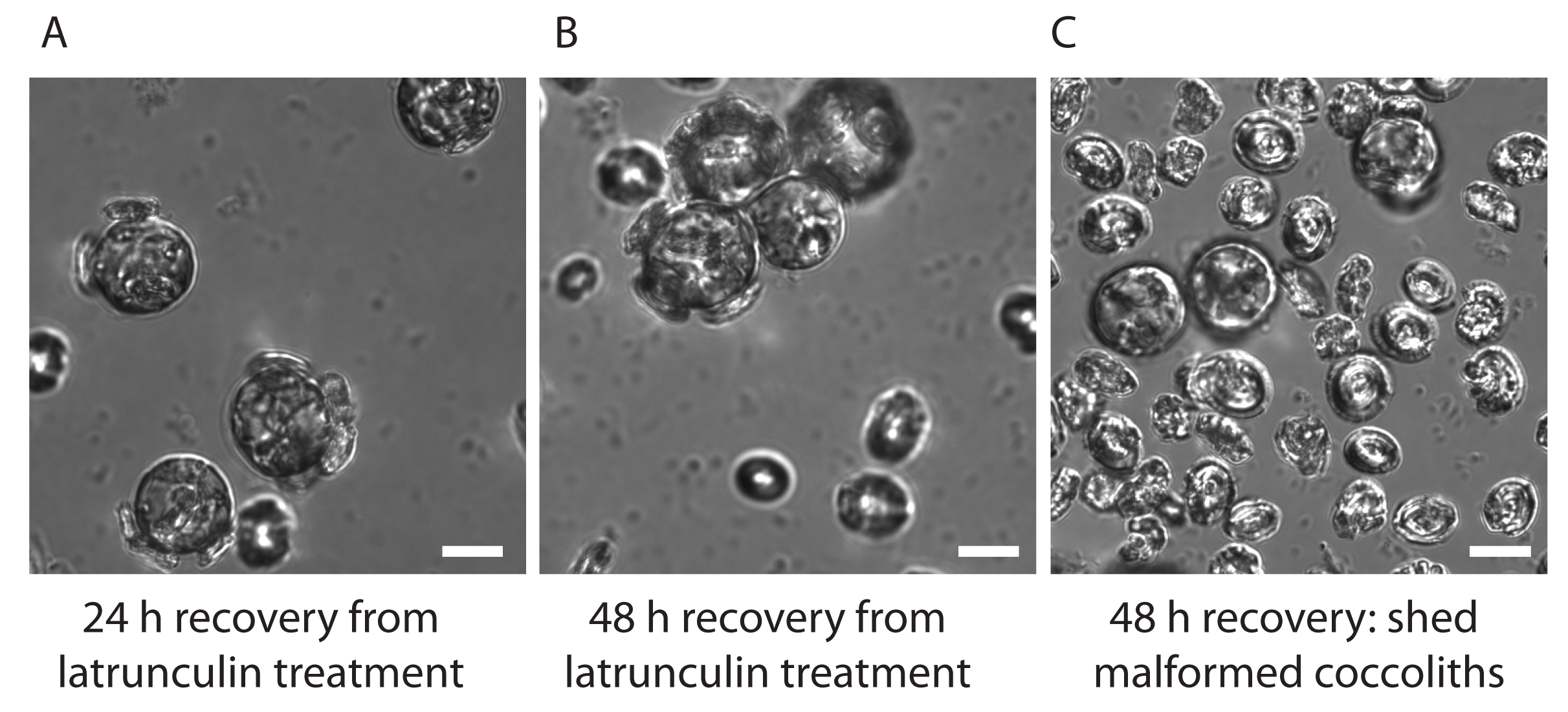


**Supplementary Figure S3: *C. braarudii* recovery from latrunculin B treatment**

DIC microscopy of *C. braarudii* cells demonstrating recovery from latrunculin B treatment. Cells were initially decalcified and then treated with 1 µM latrunculin B for 24 h. A) Cells 24 h after removal of latrunculin B. B) Cells 48 h after removal of latrunculin B. C) Large amounts of shed coccoliths present 48 h after latrunculin B removal indicated that recovering cells produced a high proportion of malformed coccoliths that were unable to attach to the cell surface. Scale bar = 5 µm.


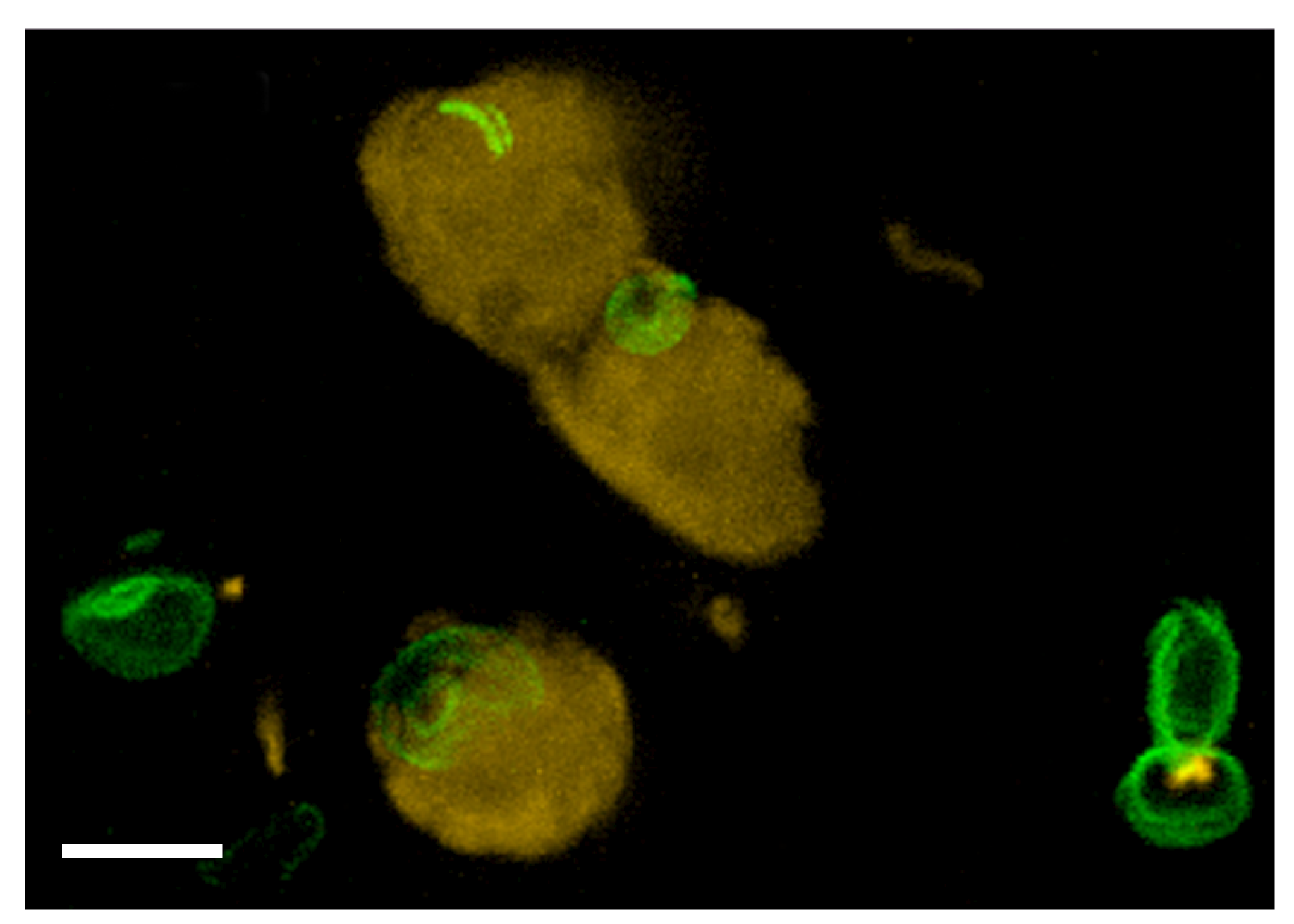


**Supplementary Figure S4: Non-specific immunofluorescence in *P. neolepis*.**

Confocal microscopy of *P. neolepis* cells demonstrating non-specific immunofluorescence. Cells were fixed and incubated with the Texas Red-conjugated secondary antibody only. A diffuse non-specific background labelling pattern can be seen in all cells (yellow), but there are none of the distinct structures that can be observed in cells treated with the anti-tubulin antibody. HCK-123 fluorescence indicating newly formed silica scales is shown in green. Scale bar = 5 µm.


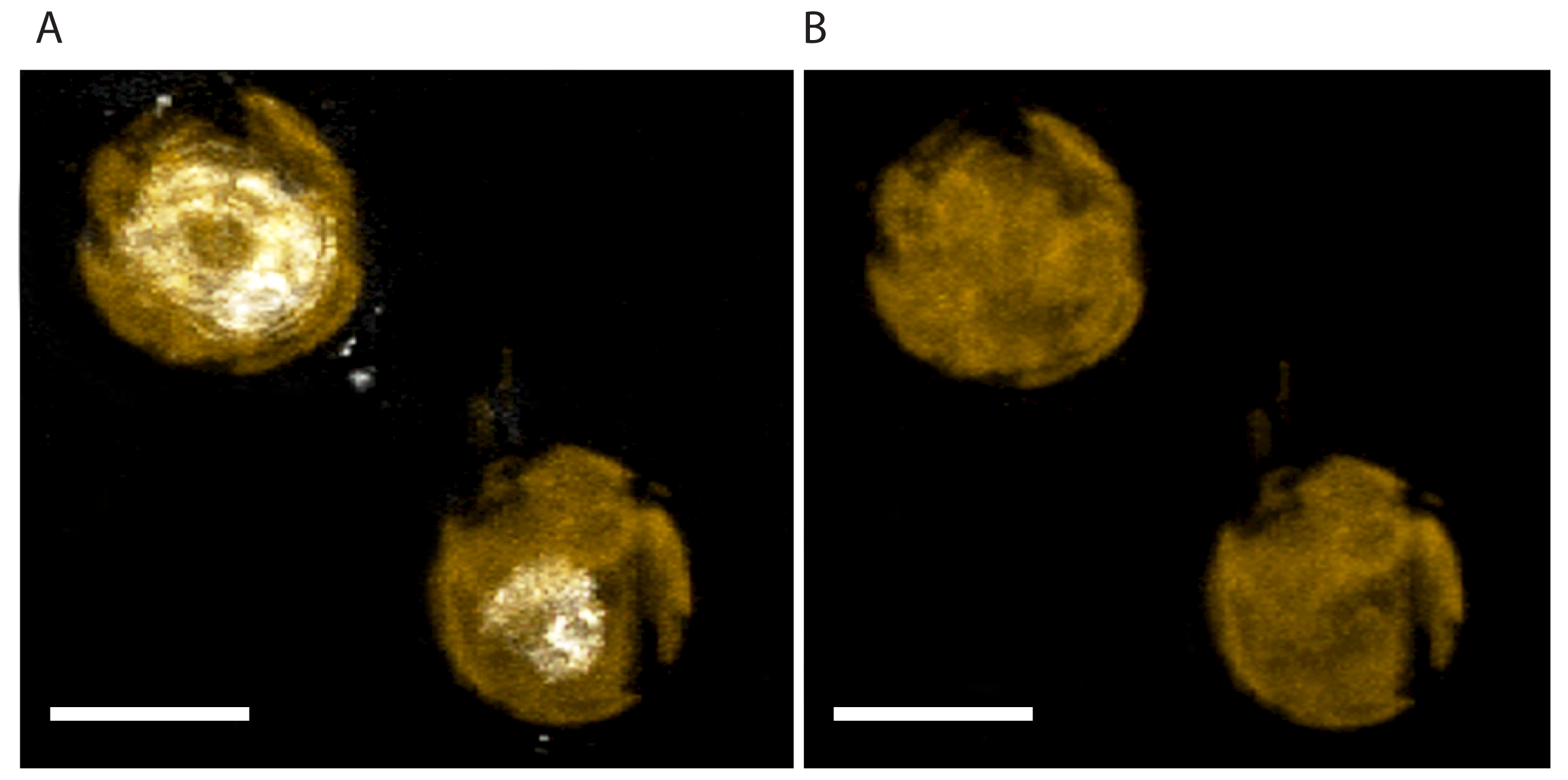


**Supplementary Figure S5: Non-specific immunofluorescence in *C. braarudii.***

Confocal microscopy of *C. braarudii* cells demonstrating non-specific immunofluorescence. Cells were fixed and incubated with the Texas Red-conjugated secondary antibody only. A diffuse non-specific background labelling pattern can be seen in all cells (yellow), but there are none of the distinct features that can be observed in cells treated with the anti-tubulin antibody. A) Cells are shown with coccolith reflectance (white) to demonstrate the position of the coccolith. B) Cells without coccolith reflectance. Scale bar = 10 µm.

**Supplementary Figure S6: Time lapse imaging of scale secretion in *P. neolepis***. DIC microscopy demonstrating secretion of silica scales by *P. neolepis* cells. Two examples are shown. The internal scales are arranged with the concave side facing the cell membrane (asterisk) and the scale is secreted in this orientation. Once secretion is completed, the orientation of the scale is then inverted (arrowed), so that the extracellular scales are positioned on the cell surface with the concave side facing the cell. Bar = 5 µm.

**Supplementary Tables**

| **Parameter** | **Control** | **DMSO_min_**  **(0.1%)** | **DMSO_max_**  **(0.35%)** | **latrunculin B**  [1 µM] | **nocodazole** [5 µg mL^-1^] |
| --- | --- | --- | --- | --- | --- |
| **Fv/Fm** | 0.73 | 0.71 | 0.71 | 0.65 | 0.71 |
| **SD** | 0.03 | 0.01 | 0.01 | 0.03 | 0.02 |
| **Cell density**  **(% of control)** | 100 | 95.5 | 93.5 | 85.4 | 86.1 |

**Supplementary Table S1: Viability measures of *P. neolepis* cells treated with cytoskeleton inhibitors for 24 h.**

Photosynthetic efficiency and cell density were measured in *P. neolepis* cells 24 h after treatment with cytoskeleton inhibitors. There is no significant change in photosynthetic efficiency with nocodazole treatment, although latrunculin causes a small decrease in mean Fv/Fm (n=3). Both treatments result in a reduced cell density after 24 h, although this is most likely due to the impact of cytoskeletal disruption on cell growth and division. DMSO treatments correspond to the lowest and highest concentration of DMSO introduced into the sample with the fluorescent dyes or the cytoskeleton inhibitors. DMSO treatment caused no difference in the photosynthetic efficiency and a very small decrease in the cell density after 24 h.

|  | **Control** | **DMSO** | **latrunculin B** | **nocodazole** |
| --- | --- | --- | --- | --- |
| **F_v_/F_m_** | 0.62 | 0.61 | 0.58 | 0.61 |
| **SD** | 0.01 | 0.01 | 0.02 | 0.01 |

**Supplementary Table S2: Photosynthetic efficiency of *C. braarudii* cells treated with cytoskeleton inhibitors for 24 h*.***

Photosynthetic efficiency was measured in *P. neolepis* cells 24 h after treatment with cytoskeleton inhibitors. There is no change in photosynthetic efficiency with nocodazole treatment, although latrunculin causes a small decrease in mean Fv/Fm (n=3).

| **Labelling** | **χ^2^ control**  **vs.**  **nocodazole** | **Df** | **p** | **Control**  **sample [%]** | **nocodazole sample [%]** |
| --- | --- | --- | --- | --- | --- |
| **None** | 9.82 | 1 | <0.05 | 42.67 | 20.00 |
| **Weak** | 0.28 | 1 | >0.05 | 45.33 | 38.67 |
| **Strong** | 53.78 | 1 | <0.05 | 12.00 | 41.33 |
| **Whole sample** | 63.88 | 2 | <0.05 |  |  |

**Supplementary Table S3: Estimation of the viability of *P. neolepis* cells treated with nocodazole using SYTOX Green.**

*P. neolepis* cells were treated with 5 µg mL^-1^ nocodazole for 24 h and then labelled with 1 µM SYTOX Green. 25 cells from triplicate analyses were imaged for each treatment and then categorised in terms in SYTOX Green labelling (Supplementary Fig S1). There was a significant increase in the number of nocodazole-treated cells exhibiting strong loading of SYTOX Green (P<0.05, Chi-squared test). This suggests that there was some reduction in membrane integrity and cell viability caused by nocodazole treatment. However, the concentration of nocodazole applied did not result in extensive cell death and the overall photosynthetic efficiency of PSII of the population was not reduced (Supplementary Table S1). We therefore conclude that the observed impacts of nocodazole on *P. neolepis* biomineralisation are not simply a consequence of a dramatic decline in the health of the population.
